# Lambeau musculocutané infra hyoidien à palette cutané horizontale pour un angiomyxome agressif de la face interne de la joue

**DOI:** 10.11604/pamj.2014.18.310.5133

**Published:** 2014-08-20

**Authors:** Mounir Kettani, Nabil Touihem, Hicham Attifi, Mounir Hmidi, Ali Boukhari, Mohamed Zalagh, Abdelhamid Messary

**Affiliations:** 1 Service d'ORL et de Chirurgie Cervico-faciale, Hôpital Militaire Moulay Ismail, Meknes, Maroc

**Keywords:** lambeau musculocutane infrahyoïdien à palette cutanée horizontale, angiomyxome agressif, tumeur mésenchymateuse, exérèse, récidive, Horizontal musculo-cutaneous infrahyoid skin pallet, aggressive angiomyxoma, mesenchymal tumor, excision, relapse

## Abstract

Décrit par Wang en 1986, le lambeau musculocutané infra hyoidien est vascularisé par l'artère thyroïdienne supérieure et comporte les muscles sternohyoïdien, sternothyroïdien et le chef supérieur du muscle Omo hyoïdien. Le prélèvement horizontal de la palette cutanée ne modifie pas la fiabilité du lambeau et permet d’éviter des cicatrices supplémentaires. L'angiomyxome agressif est une tumeur mésenchymateuse développée aux dépens du tissu conjonctif avec un site de prédilection pour les parties molles du périné féminin. Cette tumeur croit progressivement mais n'est pas métastatique. Le traitement indiqué actuellement est l'exérèse chirurgicale large. La tumeur a une tendance à la récidive locale, qui est fréquente, liée à la difficulté d'une exérèse initiale complète. Nous rapportons le cas d'un angiomyxome agressif de la joue chez un patient de 63 ans, qui a été traité par chirurgie avec reconstruction par un lambeau musculocutané infra hyoidien à palette cutanée horizontale. Les aspects cliniques, histologiques et thérapeutiques de la tumeur ont été discutés.

## Introduction

L'angiomyxome agressif est une tumeur mésenchymateuse développée aux dépens de tissu conjonctif [1]. Son site de prédilection étant les parties molles du tractus génital féminin et sa localisation cervico-faciale demeure rare [2–4]. Le problème posé par cette tumeur est surtout thérapeutique lié au risque accru de récidive après traitement [1, 2, 4]. Dans ce travail et à partir d'une observation d'un patient porteur d'un angiomyxome agressif, nous nous proposons de rappeler les aspects cliniques, histologiques et thérapeutiques de la tumeur.

## Patient et observation

**Figure 1:**
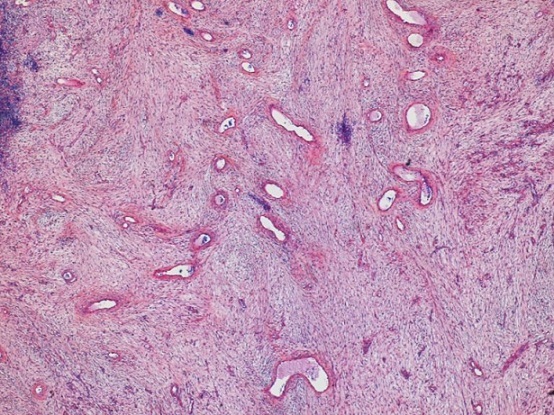
Image histologique montrant une prolifération vasculaire sur Fond myxoide, coloration HE, moyen grossissement

**Figure 2:**
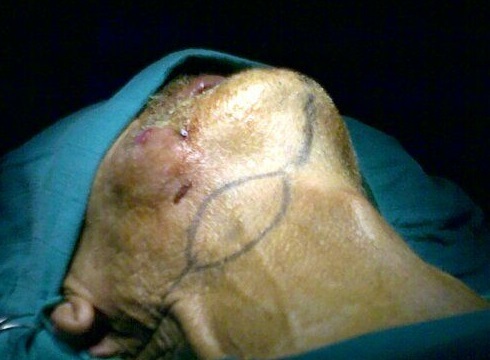
Dessin de la palette cutanée horizontale du lambeau infra hyoidien

**Figure 3:**
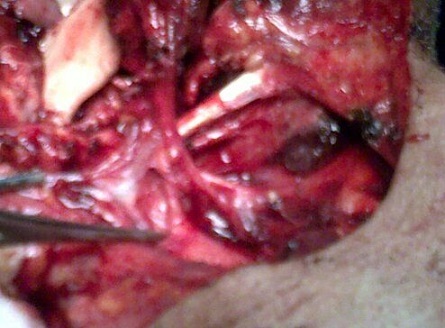
Repérage de l'artère thyroïdienne supérieure

**Figure 4:**
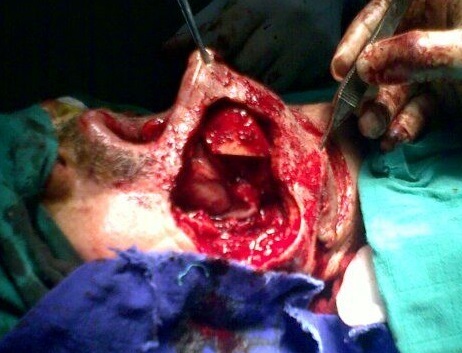
Perte de substance jugale après exérèse

**Figure 5:**
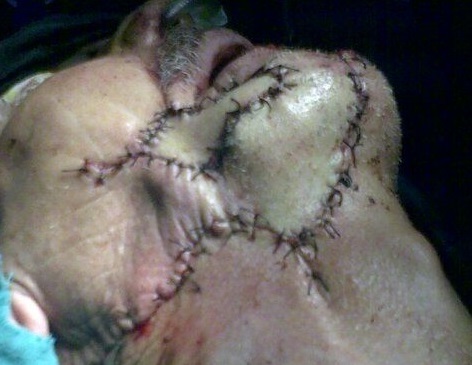
Lambeau infra hyoidien: fermeture

**Figure 6:**
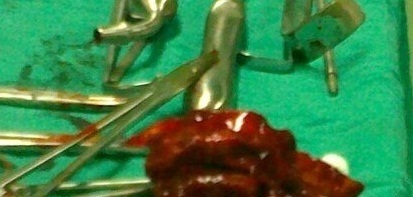
Pièce opératoire

Il s'agit d'un patient âgé de 63 ans, diabétique, hypertendu, sans habitudes toxiques, ayant consulté pour une tuméfaction de la face interne de la joue droite augmentant progressivement de volume dépuis un peu plus de 3 mois, évoluant dans un contexte de conservation de l’état général sans signes cliniques associés. L'examen clinique a objectivé l'existence d'une masse d'environ 4cm de diamètre occupant la face interne de la joue droite n'atteignant pas le trigone retro molaire, de consistance ferme et mobile par rapport aux deux plans, le reste de l'examen orl était sans particularité notamment pas de localisation synchrone au niveau de la cavité buccale et des voies aéro digestifs supérieures, les aires ganglionnaires étaient libres. Une tomodensitométrie du massif facial avait objectivé un volumineux processus expansif de la région ptérygo-palatine droite de 4 cm de grand axe. Une biopsie avec examen histopathologique avait conclu à un angiomyxome (Figure 1). Le patient a été opéré avec une exérèse large de la tumeur suivie d'une reconstruction par un lambeau infra hyoidien à palette cutanée horizontale. L'incision cutanée était centrée sur les muscles infra hyoidiens homo- latéraux à la perte de substance à hauteur de la région cricoïdienne (Figure 2), l'artère thyroïdienne supérieure repère (Figure 3), le lambeau transposé librement sur la perte de substance (Figure 4) suivi d'une fermeture en 3 plans (Figure 5). La pièce opératoire a été adressée pour examen histopathologique (Figure 6). Les suites opératoires étaient simples et le patient a quitté l'hôpital à j7. Malheureusement il a été perdu de vue.

## Discussion

Les angiomyxomes rentrent dans le cadre des tumeurs mésenchymateuses myxoïdes dont le site de prédilection est la région périnéale féminine. La localisation faciale demeure exceptionnelle [2–4]. La tumeur se présente habituellement comme une excroissance muqueuse qui croit insidieusement mais qui infiltre rapidement les tissus environnants [1]. L'incidence médiane de survenue est la 4ème décennie chez la femme (16 à 70 ans), 6ème -7ème décennies chez l'homme. Elle peut survenir également chez l'enfant. Le patient le plus jeune a 2 ans.

**Anatomopathologie:** L'angiomyxome agressif présente un aspect infiltrant plus marqué, un aspect général moins nodulaire et plus pauvre en cellules, ainsi que des cellules musculaires lisses et des fibroblastes disposés de façon concentrique autour des vaisseaux [2, 5]. Le tissu environnant apparaît myxoïde par endroits renfermant une prolifération de collagène dense [2]. Généralement, les cellules tumorales de l'angiomyxome agressif ne montrent pas d'atypies cyto-nucléaires. L’étude immuno-histochimique montre une positivité importante à la vimentine et à la désamine [1, 5]. Une étude cyto-génétique tente de démontrer que l'angiomyxome agressif serait le produit de mutation d'un gène situé sur le chromosome 12.

**Diagnostic différentiel:** Le diagnostic différentiel de l'angiomyxome se pose avec le groupe des tumeurs « stromales » à différenciation fibroblastique-Myofibroblastique [6]. De ce fait il n'est pas toujours aisé de le différencier d'un angiomyofibroblastome, d'un sarcome myxoïde de bas grade ou d'un liposarcome myxoïde [1, 2, 5–7], d'autant plus que l’étude immuno-histochimique ne permet pas toujours de trancher entre ces différentes formes histologiques pouvant présenter toutes une positivité aux marqueurs musculaires [1, 5].

**Le bilan radiologique:** L’échographie montre un aspect polyploïde hypoéchogène parfois kystique. Les images tomodensitométriques sont variables et montrent souvent une masse homogène hypodense par rapport au muscle. L'aspect en imagerie par résonance magnétique (IRM) est caractéristique montrant une tumeur iso ou hypo intense par rapport au muscle dans les séquences pondérées T1 et hyper intense dans les séquences pondérées T2. La masse se rehausse fortement et de façon hétérogène après injection de produit de contraste et peut montrer des zones moins hyper intenses au sein de la tumeur [1]. L'IRM détient un rôle important dans le diagnostic de récidive tumorale puisque les mêmes aspects radiologiques sont présents au niveau des zones de récidive [8].

**Traitement:** Dans les formes débutantes et limitées, une excision large de la tumeur est généralement le garant d'un traitement curatif avec peu de séquelles fonctionnelles [1]. Cependant, le caractère infiltrant en profondeur de l'angiomyxome rend parfois l'excision complète difficile et dangereuse. Dans tous les cas, la chirurgie demeure l'option de choix, à moins qu'elle est techniquement difficile ou que les risques encourus sont potentiels [1, 2]. Une chimiothérapie adjuvante a été tentée par certaines équipes [1]. La radiothérapie n'est pas indiquée dans ce type tumoral vue l'activité mitotique faible [1, 2]. Il est à noter qu'aucune démarche thérapeutique claire et bien codifiée n'a été jusque là précisée pour le traitement des angiomyxomes agressifs. Pour notre patient nous avons optes pour une exérèse complète de la tumeur suivie dans le même temps opératoire d'une reconstruction par un lambeau musculocutané infra hyoïdien a palette cutanée horizontale [9].

**Technique de prélèvement:** L'incision cutanée dessine un fuseau horizontal dont la partie médiane est centrée sur les muscles infra hyoidiens homo- latéraux a la perte de substance a hauteur de la région cricoïdienne. L'incision se prolonge latéralement pour l’évidement cervical. La dissection suit la description du prélèvement de Wang [10]. Le lambeau est levé de bas en haut. La veine jugulaire antérieure est liée, puis sectionnée. Les muscles sternohyoïdien et sternothyroïdien sont sectionnés près de l’échancrure sternale. La peau est suturée aux muscles a la périphérie du lambeau pour éviter le cisaillement des perforantes cutanées. Le lambeau est séparé de la glande thyroïde en passant dans l'espace avasculaire prècapsulaire. Le chef supérieur du muscle omohyoïdien est séparé de l'inférieur. Au pole supérieure de la glande thyroïde, les branches terminales de l'artère et de la veine thyroïdienne supérieure sont liées. Il faut préserver, a ce stade, la branche externe du nerf laryngé supérieur. Puis les artères thyrohyoïdienne et cricothyroïdienne sont liées. Une fois que les insertions des muscles sternothyroïdien, omohyoïdien et sternohyoïdien ont été détachées du cartilage thyroïde et de l'os hyoïde, le lambeau est transposable librement. Le site donneur est refermé sur un drain, après un éventuel évidement ganglionnaire cervical.

**Évolution:** Le caractère agressif de l'angiomyxome est dû à ses potentialités d'extension et d'infiltration muqueuse mais surtout aux récidives locales fréquentes après exérèse chirurgicale [1, 2, 4]. Ces récidives sont dues à la difficulté d'une résection tumorale complète. Elles peuvent apparaître des années après le geste chirurgical et justifient, de ce fait, la nécessité d'une surveillance régulière de ces patients [2].

## Conclusion

En conclusion, l'angiomyxome agressif est une tumeur mésenchymateuse infiltrante non métastatique. Le diagnostic anatomopathologique doit être précis afin d’éliminer une prolifération sarcomateuse maligne. Une chirurgie d'exérèse large demeure l'option thérapeutique de choix sans causer de préjudice fonctionnel important. Les récidives sont fréquentes justifiant une surveillance régulière après traitement.
